# Mindfulness, Physical Activity and Avoidance of Secondhand Smoke: A Study of College Students in Shanghai

**DOI:** 10.3390/ijerph120810106

**Published:** 2015-08-21

**Authors:** Yu Gao, Lu Shi

**Affiliations:** 1 Physical Education Department, Shanghai University of Finance and Economics, Shanghai 200433, China; E-Mail: gaoyu@mail.shufe.edu.cn; 2 Department of Public Health Sciences, Clemson University, Clemson, SC 29634, USA

**Keywords:** secondhand smoke, passive smoking, tobacco, China, mindfulness, physical activity

## Abstract

*Introduction*: To better understand the documented link between mindfulness and longevity, we examine the association between mindfulness and conscious avoidance of secondhand smoke (SHS), as well as the association between mindfulness and physical activity. *Method*: In Shanghai University of Finance and Economics (SUFE) we surveyed a convenience sample of 1516 college freshmen. We measured mindfulness, weekly physical activity, and conscious avoidance of secondhand smoke, along with demographic and behavioral covariates. We used a multilevel logistic regression to test the association between mindfulness and conscious avoidance of secondhand smoke, and used a Tobit regression model to test the association between mindfulness and metabolic equivalent hours per week. In both models the home province of the student respondent was used as the cluster variable, and demographic and behavioral covariates, such as age, gender, smoking history, household registration status (urban *vs*. rural), the perceived smog frequency in their home towns, and the asthma diagnosis. *Results*: The logistic regression of consciously avoiding SHS shows that a higher level of mindfulness was associated with an increase in the odds ratio of conscious SHS avoidance (logged odds: 0.22, standard error: 0.07, *p* < 0.01). The Tobit regression shows that a higher level of mindfulness was associated with more metabolic equivalent hours per week (Tobit coefficient: 4.09, standard error: 1.13, *p* < 0.001). *Discussion*: This study is an innovative attempt to study the behavioral issue of secondhand smoke from the perspective of the potential victim, rather than the active smoker. The observed associational patterns here are consistent with previous findings that mindfulness is associated with healthier behaviors in obesity prevention and substance use. Research designs with interventions are needed to test the causal link between mindfulness and these healthy behaviors.

## 1. Introduction

The psychological construct of mindfulness has received much academic and clinical attention in recent decades [1,2,3,4]. As a state of consciousness, which involves consciously attending to one’s moment-to-moment experience [5], the construct of mindfulness also indicates the process of active mindful noticing and distinction-making, regulating responses to potential attractors and stressors, and facilitating decision-making [1]. Higher level of mindfulness predicts favorable health outcomes and better longevity [6,7]. While the impact of mindfulness on lowering stress level [8] and improving psychological well-being [9] can be used to explain why mindful people tend to live longer, the negative association between mindfulness and overeating [10,11,12] suggest that there might exist other pathways between mindfulness and longevity. As Kristeller and Wolever [13] theorize, mindfulness-based eating awareness training [14] could reduce binge episodes and improve self-control. Psychological impact from mindfulness training such as self-control improvement might have behavioral benefits beyond reducing overeating and research on substance abuse and mindfulness suggests that mindfulness training holds promise in reducing craving and relapse episodes [15].

In this study, we test two hypotheses related with the behavioral benefits of mindfulness: first, a more mindful person might be more able to avoid health hazards, such as secondhand tobacco smoke, due to his or her well-maintained awareness of what happens at the present moment, as compared with a less mindful person who could be less aware of the ongoing body experience. Secondly, as the very concept of mindfulness implies one’s effective self-regulation [16], it is reasonable to infer that more mindful people might exert better self-regulation to conduct time-assuming and physically challenging activities, such as regular physical exercise, the kind of activities that often require a high level of impulse suppression and strong willpower. In this study, we explore these possible pathways by examining the association between trait mindfulness and conscious avoidance of secondhand smoke, as well as the association between mindfulness and physical activity among college freshmen in Shanghai, China. Below we will briefly review the literature about secondhand smoke and physical activity among college students, and then explain the rationale of our hypotheses.

We choose to explore the link between mindfulness and the avoidance of secondhand smoke because secondhand smoke exposure remains a persistent public health concern worldwide [17], particularly for vulnerable populations [18,19]. Secondhand smoke is a known risk factor of lung cancer, head and neck cancer [20], breast cancer [21,22] and cervical cancer [23]. In 2006, SHS exposure resulted in more than 42,000 deaths (including 900 infants) and an economic cost of 6.6 billion U.S. dollars due to lost productivity [24]. In the United States, the annual death toll from SHS is more than twice the number of AIDS deaths [25], and the economic cost from SHS is more than twice that from the combination of melanoma and non-melanoma skin cancer [26]. As the world’s largest tobacco market, China had a smoking prevalence around 52.9% among men and 2.4% among women in 2010 [27]. As estimated in 2005, an annual total of 673,000 deaths in China were attributable to tobacco smoking [28]. This number may still be a serious underestimate since the high prevalence of passive smoking was not accounted for. Airborne nicotine was detected among 91% of the sampled public indoor environments [29], making the vast majority of the Chinese people potential victims of passive smoking. While smoking ban in public indoor environments has been implemented in many Asian countries, it is likely to take a very long time before a nationwide smoking ban to effectively minimize tobacco smoke from public indoor environment [30,31], and even then household secondhand smoke exposure as a very significant source of secondhand smoke is likely to still persist to harm the health of nonsmokers. Finally, a “quitter” living in an environment of high smoking prevalence might himself or herself suddenly become a victim of secondhand smoke [32] as most people close to him or her are likely to smoke. Thus, the benefits attainable from smoking cessation are compromised in communities with high smoking prevalence.

Therefore, given what we know about the morbidity and mortality outcomes of secondhand smoke exposure, it is important for a nonsmoker to consciously avoid secondhand smoke to reduce the harm from secondhand smoke exposure [33], and the deliberate avoidance of secondhand smoke could require a high level of trait mindfulness: a less mindful person might be too immersed in thoughts about the past and the future to be aware the existence of secondhand smoke in the environment, and, thus, might be less likely to take action to stay away from the tobacco smoke than a more mindful person.

Our rationale in making this hypothesis is in accordance with the documented definition of mindlessness: “the human tendency to operate on autopilot without concern for consequences or outcome, whether by stereotyping, performing mechanically or simply by not paying attention.” [34] Hanan *et al.* [35] inferred from an extended theoretical framework of Theory of Planned Behavior that the mental state of being mindful could help a driver “be aware of potential risks which may change instantly.” This observation is supported by the negative association between mindfulness and texting while driving among young adult drivers [36]. Similarly, we infer that a nonsmoking person with higher level of trait mindfulness could be quicker to detect the secondhand smoke around and take action to avoid it.

Our second hypothesized pathway between mindfulness and longevity is through time spent on physical activity: people with higher level of mindfulness might be able to make more time available for physical activity, which leads to lower mortality in life as we observed from documented evidence [37]. This hypothesis is derived from the observed link between mindfulness and self-regulation [16]. Increasing physical activity could require a high level of self-regulation to overcome the instinct of falling back to more effortless behavior, such as viewing television, playing video games, and surfing the Internet. Thus, we infer that people with a higher level of trait mindfulness, with a higher level of self-regulation, might be more likely to be able to overcome the temptation of physical inactivity than those who are more mindless with a lower level of self-regulation.

Using a sample of college students in China, we aim to examine this hypothesized link between physical activity and trait mindfulness as well as our hypothesized link between one’s trait mindfulness and his or her conscious avoidance of secondhand smoke. If such links are confirmed, we might be able to better understand the comprehensive health benefits of mindfulness interventions: a person with increased mindfulness could become more physically active and be more likely to avoid environmental hazards like secondhand smoke.

## 2. Method

With the institutional approval from Shanghai University of Finance and Economics (SUFE), in December 2014 we collected a convenient sample of 1516 college freshmen, who participated in this survey at the end of their Fall semester on a voluntary basis. The timing was chosen to capture their period of transitioning to the college life when there might be more of a difference between the time management style of those more mindful and those more mindless.

Shanghai University of Finance and Economics is one of the most selective colleges in China, and we are aware that its selectiveness could threaten the external validity of our study. However, SUFE’s selectiveness could also mean that there is a certain level of homogeneity among the study participants in terms of their scholastic achievement, thus variations in unmeasured variables are less likely to bias the estimation of key associations in question.

Our survey questionnaire contains a total of 110 questions, including several validated scales that operationalize the measurement of our key variables. For example, to measure the construct of mindfulness our survey used a well validated scale that consisted of 25 questions: mindful attention awareness scale [38]. We tested the Cronbach’s alpha for the MAAS and obtained a value of 0.85, which means that the questionnaire items share a good internal consistency for this sample of Chinese college freshmen. To measure the students’ physical activity, we used the Chinese version of short-form international physical activity questionnaire [39] and then code the responses into a variable that captures the respondent’s weekly metabolic equivalent hours.

We measure the respondent’s conscious avoidance of secondhand smoke by asking the respondent “when you find that someone near you is smoking tobacco products, do you deliberately stay away from the tobacco smoke to avoid inhaling harmful substance?” The respondent was given five options: 1. Never; 2. Seldom; 3. Sometimes; 4. Often. 5. Always. We recode those choosing “often” or “always” as 1 and those choosing “never,” “seldom,” or “sometimes” as 0, creating a dummy variable for conscious avoidance of secondhand tobacco smoke.

We examine the association between mindfulness and conscious avoidance of secondhand smoke by using a logistic regression with robust standard errors (the student’s home province as the cluster variable), and we control for gender, age, smoking history, the frequency of smog in the hometown, whether the respondent had a diagnosis of asthma and whether the respondent came from an urban background.

The same set of covariates was also used in our model about mindfulness and weekly metabolic equivalent hours, where we follow the procedure of converting metabolic equivalent hours using IPAQ for a comprehensive measurement of physical activity [40]: 3.3 METs for time spent on walking, 4 METs for time spent on moderate activity, 8 METs for time spent on vigorous activity (www.ipaq.ki.se). As for the model selection, we adopt a left-censored Tobit regression [41] to account for the fact that 15.44% of our sample had less than one metabolic equivalent hour of physical activity per week.

## 3. Results

**Figure 1:**
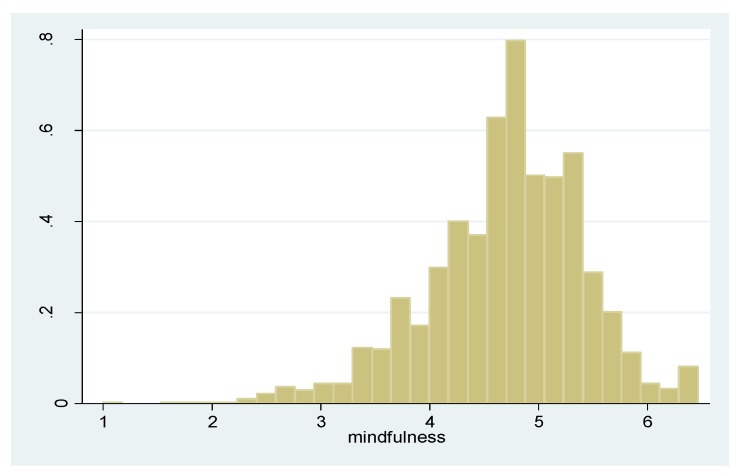
The Histogram of the Mindful Attention Awareness Scale.

**Figure 2:**
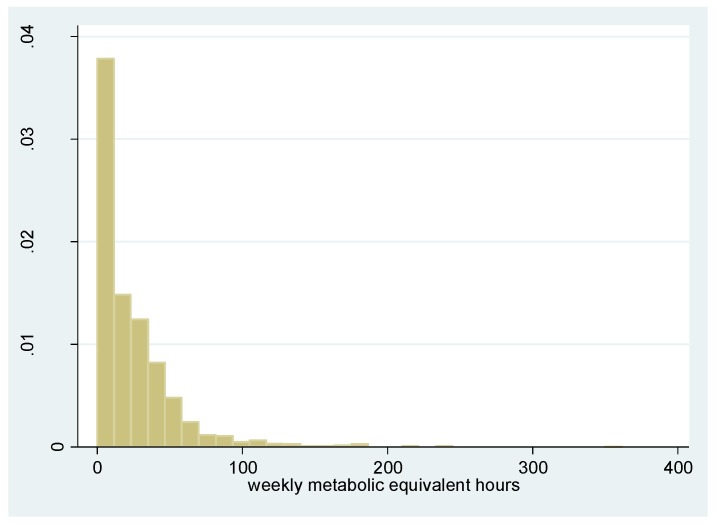
The Histogram of the Weekly Metabolic Equivalent Hours.

The mindful attention awareness scale ranges from 1 to 6.467 with a mean value of 4.701 (Figure 1). The weekly metabolic equivalent hours are 24.01 on average, ranging from 0 to 361.7 (Figure 2). Table 1 charts the descriptive statistics of our study sample. After deleting cases with missing values in variables used in this analysis, we were left with an analysis sample of 1354 cases. Male students constituted 38.0% of the study sample. The average age was 18.30 and 81.6% had a registered residential status in Urban China. Around 3.5% of the respondents had been diagnosed as having asthma in the past, while 37.3% reported having smog in their hometown “often,” “very often,” or “daily.” Around 23.8% of the students reported having ever smoked cigarettes and 75.8% reported “often” or “always” consciously avoiding secondhand smoke. The average number of metabolic equivalent hours in this analysis sample is 24.9 per week.

**Table 1:** Descriptive statistics of the sample from Shanghai University of Finance and Economics.

	Frequency	Mean
Metabolic equivalent hours per week (MET hours)	-	24.01
Consciously avoiding secondhand smoke	75.8%	-
Mindful Awareness and Attention Scale	-	3.70
Male	38.0%	-
Ever smoked	23.8%	-
Smog at hometown	-	-
Rare	22.3%	-
Sometimes	40.6%	-
Often	31.6%	-
Very often	5.4%	-
Daily	0.4%	-
Urban background	81.6%	-
Diagnosed with asthma	3.5%	-
Age	-	18.30
*N*	1354	

**Table 2:** Logistic regression of consciously avoiding secondhand smoke and Tobit regression of physical inactivity.

	Avoid Secondhand Smoke		Metabolic Equivalent Hours Per Week	
	Logged Odds	Standard Errors	Tobit Coefficients	Standard Errors
Mindfulness	0.22 ^**^	(0.07)	4.09 ^***^	(1.13)
Male	−0.55 ^***^	(0.13)	12.56 ^***^	(2.72)
Ever smoked	−0.14 ^*^	(0.06)	−0.16	(1.38)
Smog at hometown (“rare” as the referent group)				
*Sometimes*	0.58 ^***^	(0.17)	5.50 ^**^	(2.11)
*Often*	0.60 ^**^	(0.22)	6.59	(3.69)
*Very often*	0.47 ^*^	(0.21)	4.65	(5.00)
Urban background	0.25	(0.15)	−0.42	(2.59)
Diagnosed with asthma	0.02	(0.16)	−4.63	(6.19)
Age	−0.17 ^*^	(0.08)	−0.39	(1.54)
*N*	1354		1354	

Standard errors in parentheses; ^*^ *p* < 0.05, ^**^ *p* < 0.01, ^***^ *p* < 0.001.

The logistic regression of consciously avoiding secondhand smoke (Table 2) shows that a higher level of mindfulness was associated with an increase in the odds ratio of conscious SHS avoidance (logged odds: 0.22, standard error: 0.07, *p* < 0.01). This means that one unit increase in mindfulness scale was associated with a statistically significant increase in the odds ratio of consciously avoiding secondhand smoke (Odds ratio = 1.25, standard error = 0.09). Other significant and positive predictors of consciously avoiding secondhand smoke include self-reported frequency of smog in one’s hometown: as compared with the reference group of “never” and “rarely,” all three categories of smog frequency were positively associated with consciously avoiding secondhand smoke (for “sometimes,” logged odds: 0.58, standard error: 0.17, *p* < 0.001; for “often,” logged odds: 0.60, standard error: 0.22, *p* < 0.01; for “very often,” logged odds: 0.47, standard errors: 0.21, *p* < 0.05). Negative and statistically significant predictors of consciously avoiding secondhand smoke include the male gender (logged odds: −0.55, standard error: 0.13, *p* < 0.001), experience of having smoked cigarettes (logged odds: −0.14, standard error: 0.06, *p* < 0.05) and self-reported age (logged odds: −0.17, standard error: 0.08, *p* < 0.05).

The Tobit regression of weekly physical activity (Table 2) shows that a higher level of mindfulness was associated with more metabolic equivalent hours per week (Tobit coefficient: 4.09, standard error: 1.13, *p* < 0.001). The two other significantly positive predictor is the male gender (Tobit coefficient: 12.56, standard error: 2.72, *p* < 0.001) and the “sometimes” category when the respondent is asked about the frequency of smog in his or her hometown.

## 4. Discussion

Studies about mindfulness and abstinence from substance abuse and studies about mindless eating suggest that the documented link between mindfulness and longevity might not solely rely on the pathway of improving biomarkers such as decreasing cortisol level [42] and lowering blood pressure [43]. It is possible that mindfulness could be associated with healthier behaviors which lead to a longer and healthier life. Our exploratory study supports our hypothesized pathways between mindfulness and longevity: those more mindful people are more likely to avoid health hazards, such as secondhand smoke and they report a higher amount of metabolic equivalent hours per week. The observed associational patterns here are consistent with previous findings that mindfulness is associated with stronger self-regulation [16], which enables people to accomplish possibly challenging tasks, such as maintaining a regular schedule for a sufficient amount of physical activity.

However, given the cross-sectional and observational nature of our data we cannot assert with certainty that mindfulness causes these protective behaviors among younger adults. We cannot rule out the recall bias mechanism whereby those people with higher level of trait mindfulness might be more likely to recall the instances of avoiding secondhand smoke, or the actual physical activity they finished. Meanwhile, the fact that the dataset is based on the selective school of SUFE also compromises the generalizability of our findings. Finally, our measurement of both physical activity and secondhand smoke avoidance is based upon the respondent’s self-report. For future studies looking for stronger internal validity, researchers will need to collect biomarkers, such as cotinine for tobacco exposure, and use equipment, such as accerometers and/or pedometers for physical activity measurements.

We are also aware that a mechanism of reverse causality could be behind our observed association between mindfulness and physical activity: physical activity helps a person become more mindful. While we cannot rule out this competing hypothesis with the data we have, what we measure here is the weekly physical activity among college freshmen who had just established their weekly schedule within the past few months, and there is no solid evidence that physical activity in high school is highly correlated with physical activity in college. Meanwhile, the questionnaire we used to measure trait mindfulness is a measure of mindfulness as reflected by cognitive and behavioral experiences of multiple years. It is unlikely that a relatively new physical activity schedule could have contributed significantly to one’s trait mindfulness of the past several years. .

As we plan for a longitudinal follow-up with these college freshmen in Shanghai University of Finance and Economics, we are aware that experimental designs with participants randomized to the mindfulness intervention are needed to examine the causal link between mindfulness and environmental hazard avoidance, as well as the causal link between mindfulness and physical activity. An experimental design with mindfulness intervention will not only offer better evidence for the link between mindfulness and health behavior but also have stronger public health relevance if the results support a causal mechanism: we might be able to use mindfulness intervention as a way to boost more physical activity and help avoid environmental health hazard.

## 5. Conclusions

To the best of our knowledge, this is the first study that uses a large sample of more than one thousand younger adults to explore the link between trait mindfulness and health behavior such as avoiding secondhand smoke and physical activity. As this is the first wave of a longitudinal study of mindfulness and cognitive-behavioral health among college students, the concern that our study is based upon a cross-sectional dataset will be partially addressed once we collect longitudinal data from these respondents. We expect to examine the comprehensive long-term impact of trait mindfulness using follow-up waves of data.
